# Poller Screws and Post-Operative Pain Relief in Tibial Fractures

**DOI:** 10.7759/cureus.54811

**Published:** 2024-02-24

**Authors:** Florence Bradshaw, Edward Wakefield, James Zhang, Ansh Tandon, Matija Krkovic

**Affiliations:** 1 School of Clinical Medicine, University of Cambridge, Cambridge, GBR; 2 Orthopaedics, Addenbrooke's Hospital, Cambridge University Hospitals NHS Foundation Trust, Cambridge, GBR; 3 Trauma and Orthopaedics, Addenbrooke's Hospital, Cambridge University Hospitals NHS Foundation Trust, Cambridge, GBR

**Keywords:** orthopaedics surgery, intramedullary nails, tibial fractures, pain, poller screw

## Abstract

Introduction

Poller screws optimise fracture alignment in those fractures managed with intramedullary (IM) nails. They enhance stability, control nail insertion, and prevent translation. Indications encompass acute fractures, delayed unions/non-unions, and deformity. Classified into four generations, they've shown positive outcomes: improved alignment, reduced complications, and shorter healing. However, their pain management impact is understudied. This retrospective cohort study aimed to compare opioid medication needs in tibial fractures managed with IM nails with and without poller screws.

Methods

Between January 2015 and December 2022, a retrospective analysis was conducted on tibial fractures treated at a major trauma centre. Patients primarily treated with IM nails were included. Patient and operation notes as well as radiographs, were reviewed to identify poller screw utilisation. Opioid medication data was collected and converted to “coverage” (days) and “strength” (morphine milligrams equivalent or MME). Two-tailed independent samples T-tests were performed to determine differences between patients treated with (n=205) and without poller screws (n=540).

Results

Patients with poller screws had fewer days with opioid prescriptions in the second post-operative month (6.8 vs. 8.9 days, p=0.038) and significantly lower opioid strength requirements across the first post-operative year (688.4 vs. 1295.4 MME, p=0.001), except the first month.

Conclusion

There is limited research on the connection between poller screws and pain. This study discusses their potential to reduce post-operative pain in tibial fractures. The results highlight the importance of using poller screws alongside IM. This combination appears to be effective in improving post-operative pain management and enhancing overall patient outcomes.

## Introduction

Poller screws, also known as blocking screws, play a crucial role in the field of orthopaedics by optimizing the alignment of fractures that undergo treatment with intramedullary (IM) nails [1]. The name "Poller" draws an analogy from the road bollards utilized to direct or obstruct traffic flow [2]. In the context of orthopaedics, these screws function as a barrier, impeding the potential translation and rotation of the IM nail within the medullary canal [3].

The indications for poller screws include correcting alignment after nail insertion, maintaining or improving stability of the bone-implant complex, controlling the nail during insertion, and preventing translation of the nail along coronally placed locking screws [1]. Their usage is prevalent in acute fractures, delayed unions, and instances of malalignment arising from prior external fixation or IM nailing treatments [1].

First-generation screws are inserted to create a corridor inside the bone for the IM nail. Second-generation screws utilize the elastic characteristics of the IM nail to compress fractures by deflecting the nail inside the fragments, positioned on a single side of the fracture. In the third generation, two screws are used in total. Screws are inserted on both sides, adhering to the principles of the second generation [4]. In fourth-generation screws, four screws are used. Two screws are used in the anterior-posterior plane and two in the lateral plane.

Studies have reported positive outcomes with the use of poller screws to manage fractures treated with IM nails, including improved fracture alignment, reduced complication rates such as non-union and malunion, shortened healing time, and improved clinical outcomes [3, 5].

While poller screws are primarily used to enhance stability and alignment in fractures treated with IM nails [1], their specific impact on pain management has not been extensively studied. Pain management in orthopaedic fractures involves a comprehensive approach that considers various factors, and the use of poller screws may contribute to pain management by improving fracture stability. Further research is needed to investigate the specific effects of poller screws on pain outcomes in orthopaedic fractures. This retrospective, observational cohort study aimed to investigate the differences in pain medication requirements for tibial fractures treated with and without poller screws.

## Materials and methods

Between January 2015 and December 2022, we reviewed all tibial fractures treated at a major trauma centre. Patients whose primary fixation modality was IM nail were included in the study. Exclusion criteria were patients who did not receive follow-up appointments at the same hospital due to living in another region, lost to follow-up after discharge from the operation, or died from non-orthopaedic-related injuries from the initial trauma or other conditions, within one month post-operatively.

For each patient in the cohort, we reviewed their radiographs and operation notes to determine whether they received poller screws in their fixation. We also collected additional data on the age at admission and gender of the patient. The poller screw group was investigated against the control group without poller screws. Within the poller screw group, the type of poller screw received was further subdivided into first, second, third, and fourth-generation screws, to undergo subgroup analysis.

To evaluate the opioid medications prescribed to patients in the first year after their injury, we retrieved data on all medication types and converted them into morphine milligrams equivalent (MME) using the Centers for Disease Control and Prevention opioid conversion factors [6]. We calculated the total opioid strength and coverage for each patient using the start and end date of each medication. To create a timeline of the rehabilitation period, we used Python code, which allowed us to combine the raw opioid data and determine the overall strength of MME and the number of days the patient was prescribed at least one opioid medication. We divided the data into one-month blocks until the sixth post-injury month and combined the seventh to 12th post-injury months.

We defined the term "coverage" as the number of days the patient was prescribed at least one opioid medication, indicating the background level of pain experienced over the specific time period that could not be controlled by non-opioid means. The term "strength" refers to the total dose of MMEs prescribed to the patient over a specific time period, representing the severity of overall pain. Higher levels of pain require a higher combined dosage in addition to widespread coverage [7].

We calculated the overall comparison between patients treated without poller screws and those treated with poller screws, for both coverage and strength, over each individual time period, as well as the aggregate first post-operative year interval. We conducted a two-tailed independent samples T-test to analyse for any statistically significant differences, with the significance level set at p<0.05.

We also calculated the t-test comparison between each generation of poller screw versus the non-poller screw cohort, to ensure the detection of any differences of generations. Finally, within the poller screw cohort, for each generation of screws, we performed the same test to elucidate differences between the generations in opioid requirements. All statistical analysis was conducted using the software IBM SPSS v28 (IBM Corp., Armonk, NY, USA).

## Results

A total of 745 patients were included in this study, with 205 patients receiving poller screws and 540 not receiving poller screws. The entire cohort’s demographic details are displayed in Table 1.

**Table 1:** Demographic breakdown and injury characteristics of the total cohort including the number of patients in the poller screw and non-poller screw groups.

	Entire cohort	Poller screw patients	Non-poller screw patients
Number of patients	745	205	540
Age (years)	41.75	44.71	40.63
Days in Hospital	18.52	11.80	21.08
Male	468	132	336
Female	277	73	204

On analysis of each subinterval in the first post-operative year, the coverage and strength requirements for opioids are shown in Table 2, with the P-value of the T-test difference between poller screw and non-poller screw patients displayed.

**Table 2:** The coverage (days) and strength (MME) breakdown for poller screw and non-poller screw patients in the sub-intervals for the first post-operative year, with the result from two-tailed T-test displayed as the P-value. Those results marked with * indicate significance at p<0.05. MME = morphine milligrams equivalents

Outcome and time interval	Poller screw patients	Non-poller screw patients	P-value
Coverage (days)			
Month 1	14.345	15.272	0.266
Month 2	6.833	8.939	0.038*
Month 3	5.099	6.400	0.166
Month 4	4.404	5.470	0.230
Month 5	3.901	5.070	0.170
Month 6	3.443	4.485	0.198
Months 7 - 12	15.645	21.902	0.138
First year	54.234	67.539	0.126
Strength (MME)			
Month 1	235.939	286.695	0.280
Month 2	78.430	176.469	0.000*
Month 3	59.124	130.461	0.003*
Month 4	50.683	107.015	0.015*
Month 5	44.409	91.224	0.005*
Month 6	37.906	77.055	0.009*
Months 7 - 12	168.473	426.508	0.006*
First year	668.380	1295.425	0.001*

For the coverage requirements, patients who received poller screws had statistically significantly fewer days (6.8 days) requiring at least one opioid medication prescription, compared to patients without poller screws (8.9 days) (p = 0.038), in the second post-operative month, however, all other time periods did not reach significance despite higher mean days of coverage for the non-poller screw group.

For opioid strength requirements, apart from the first post-operative month, every time period had patients with poller screws requiring lower strength of opioids than those without poller screws at a statistically significant level, requiring almost half the amount across the entire year (688.4 MME vs. 1295.4 MME).

The comparison between each of the four generations of poller screws individually with the non-poller screw cohort is shown in Table 3. There were no first-generation poller screws used at our hospital over the time period of the study. Within the poller screw group, the generation of poller screw was documented, and subgroup analysis performed within the cohort to determine relative opioid requirements between generations. The results are shown in Table 3.

**Table 3:** Comparisons of opioid requirements, for strength and coverage outcomes, for each generation of poller screw with the non-poller screw cohort and in poller screw treated fracture patients, between each generation of device. Those results marked with * indicate significance at p<0.05. MME = morphine milligrams equivalents

	Generation of Screw			
	No screw	2nd generation	3rd generation	4th generation
Total number of screws	540	119	41	45
1st-year coverage (days)	64.28	61.82	41.70	45.84
P-value 1st-year coverage (with no screw)	-	0.82	0.079	0.114
P-value 1st-year coverage (within generation)	-	0.10	0.33	0.482-
1st-year strength (MME)	1185.14	795.32	520.58	467.33
P-value 1st-year strength (with no screw)	-	0.045*	<0.001*	<0.001*
P-value 1st-year strength (within generation)		0.034*	0.284	0.111

We found a statistically significant difference in outcome only for second-generation screws, with statistically significantly higher opioid strength requirements compared to the other generations at the first-year post-operative time interval level.

## Discussion

**Figure 1:**
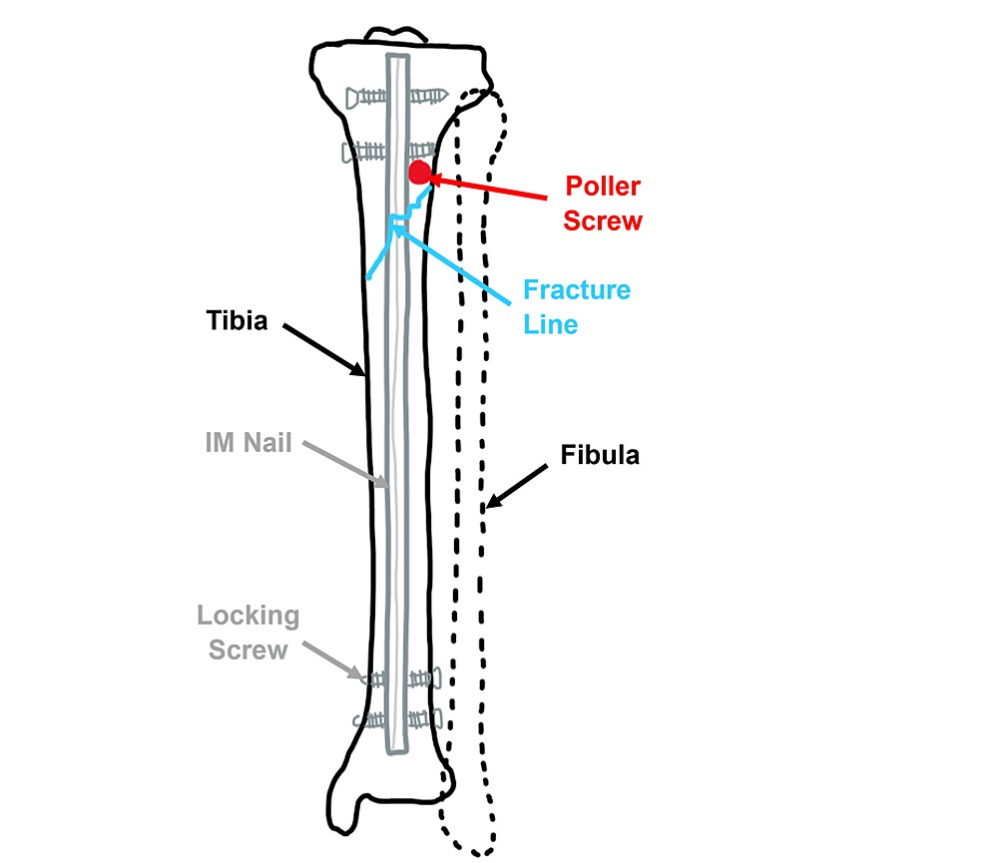
A diagram showing the use of a poller screw alongside an intramedullary (IM) nail in a tibial fracture.

In the literature, there is limited information available regarding the directional relationship between poller screws and pain. In a study by Krettek et al., the clinical use of poller screws to supplement stability after fixation with statically locked IM nails in tibial fractures was evaluated. However, the study did not specifically focus on pain outcomes. The indications for using poller screws in this study included correcting alignment after nail insertion, maintaining or improving stability of the bone-implant complex, and controlling the nail during insertion [1]. Figure 1 shows how poller screws can be used alongside IM nails in tibial fractures.

While these factors may contribute to pain management by improving fracture alignment and stability, the study did not provide specific data on pain outcomes.

Our study aimed to elucidate if the use of poller screws could reduce pain in those tibial fractures managed with IM nails. The research spanned an extended timeframe and encompassed a total of 745 patients, out of which 205 received poller screws. In comparison to the known existing literature that discusses the use and impact of poller screws [2], our study has the largest cohort of cases involving poller screws. Importantly, it stands alone as the sole investigation into the correlation between poller screw utilisation and pain reduction.

It is noteworthy that much of the literature on this topic comprises case series rather than retrospective observational cohort studies [2]. Our study, in line with other research on poller screws, maintains a similar average patient age of 41.75 years [2].

The observation of significantly lower opioid strength requirements among patients with poller screws compared to those without, coupled with the trend of decreased opioid coverage (albeit not statistically significant), offers valuable insights into the potential role of poller screws in mitigating post-operative pain in fracture management. This finding holds particular significance for patients receiving opioids, as it addresses the concerns of addiction and adverse effects associated with these medications [8].

It is important to note that pain management in orthopaedic fractures involves a multifactorial approach, including appropriate analgesic medications, immobilization, physical therapy, and patient-specific factors. The use of poller screws may play a role in improving fracture stability, which can indirectly contribute to pain management by facilitating proper healing and reducing the risk of complications.

Four different generations of poller screws exist, which are selected based on the surgeon's discretion and judgment of forces acting on the fracture [4]. When splitting patients who received poller screws into each generation and comparing them with those who underwent IM nail management for their tibial fractures alone, recipients of poller screws exhibited significantly reduced opioid strength requirements across all generations. However, this trend was not observed in terms of opioid coverage.

Intriguingly, when comparing the poller screw generations against each other, it became apparent that second-generation poller screws required significantly higher opioid strength requirements to the rest of the poller screw cohort. This discrepancy could potentially be attributed to the specific utilisation of second-generation poller screws, which are employed on a single side of the fracture [4]. This localised application might induce heightened forces on one side of the fracture, thereby potentially leading to elevated pain requirements compared to other generations of poller screws.

It should be considered that the distribution of poller screws among the different generations in our sample is not uniform. No first-generation poller screws were used in the time period of the study. Generation three, and four exhibit significantly smaller sample sizes of poller screws in comparison to generation two. This uneven distribution has the potential to impact the observed results, introducing a degree of variability into the analysis.

The retrospective nature of our study limits us from directly assessing patients' actual opioid consumption; we can only comment on their prescribed dosages and regimens. Our analysis is based solely on prescription records rather than the patients' self-reported opioid intake. Nevertheless, the presence of prescription records spanning multiple months suggests some degree of actual patient opioid consumption, and there is no evidence to suggest different adherence rates between poller and non-poller screw patients, therefore the trends should be preserved.

It's important to emphasize that our investigation exclusively focused on the utilization of poller screws in tibial fractures managed with IM nails. Exploring the impact of poller screws on pain of the management fractures in different bones, such as the femur, would be of great interest. Additionally, we could integrate "patient-reported outcome measures" (or PROMs) which evaluate pain perceptions and other factors over time through follow-up appointment questionnaires, into future studies which hold promise to further enhance the robustness of our findings.

Finally, as mentioned previously, we appreciate choice of surgical fixation metalwork hardware is only one aspect of a complex web of intervention and rehabilitation for the patient, and there are countless factors that will influence the final level of opioids consumed. However, we have presented a comparison based on one factor, with all other factors likely comparable between the two cohorts, as rehabilitation regimes do not differentiate between these two similar therapies.

Despite the limitations, this paper provides an initial insight into the impact that poller screws have on pain outcomes in tibial fractures managed with IM nails, however further research is needed to further investigate this effect and if poller screws should be used alongside IM nails more frequently.

## Conclusions

The connection between poller screws and pain is understudied, however, this study highlights the potential impact of poller screws on post-operative pain management through reduced opioid requirements, with the use of a large cohort with detailed breakdown of rehabilitation opioid requirements. The findings suggest that poller screws enhance pain control when used alongside IM nails in the treatment of tibial fractures.
